# On thresholds for controlling negative particle (PM_2.5_) readings in air quality reporting

**DOI:** 10.1007/s10661-023-11750-4

**Published:** 2023-09-12

**Authors:** Ningbo Jiang, Rinat Akter, Glenn Ross, Stephen White, John Kirkwood, Gunaratnam Gunashanhar, Scott Thompson, Matthew Riley, Merched Azzi

**Affiliations:** Science, Economics and Insights Division, New South Wales Department of Planning and Environment, Sydney, Australia

**Keywords:** Fine particle (PM_2.5_) monitoring, Beta Attenuation Monitor (BAM), Negative reading, Data validation, Compliance reporting

## Abstract

Ambient PM_2.5_ (particles less than 2.5 μm in diameter) is monitored in many countries including Australia. Occasionally PM_2.5_ instruments may report negative measurements, although in realty the ambient air can never contain negative amounts of particles. Some negative readings are caused by instrument faults or procedural errors, thus can be simply invalidated from air quality reporting. There are occasions, however, when negative readings occur due to other factors including technological or procedural limitations. Treatment of such negative data requires consideration of factors such as measurement uncertainty, instrument noise and risk for significant bias in air quality reporting. There is very limited documentation on handling negative PM_2.5_ data in the literature. This paper demonstrates how a threshold is determined for controlling *negative hourly* PM_2.5_ readings in the New South Wales (NSW) air quality data system. The investigation involved a review of thresholds used in different data systems and an assessment of instrument measurement uncertainties, zero air test data and impacts on key reporting statistics when applying different thresholds to historical datasets. The results show that a threshold of −10.0 μg/m^3^ appears optimal for controlling negative PM_2.5_ data in public reporting. This choice is consistent with the measurement uncertainty estimates and the zero air test data statistics calculated for the NSW Air Quality Monitoring Network, and is expected not to have significant impacts on key compliance reporting statistics such as data availability and annual average pollution levels. The analysis can be useful for air quality monitoring in other Australian jurisdictions or wider context.

## Introduction

In air quality management, particulate matter (PM), also known as particles or particle pollution, refers to a mixture of solid particles and liquid droplets suspended in the air. Studies have shown that particle pollution has significant impacts on the environment and human health (OECD, 2016; WHO, 2013). For regulatory purposes, ambient particle pollution is often monitored as PM_10_ and PM_2.5_, i.e. particles less than 10 μm and 2.5 μm in diameter, respectively. PM_2.5_ can be transported further and persist longer in the atmosphere than PM_10_ (Keywood et al., 2016). PM_2.5_ pollution is of greater public concern in many countries including Australia (DPIE, 2021; OECD, 2016), as the particles are small enough to penetrate deeply into human lungs and bloodstreams, causing adverse health effects such as increased respiratory symptoms, heart problems and premature deaths (Anderson et al., 2012; Broome et al., 2020).

**Fig. 1:**
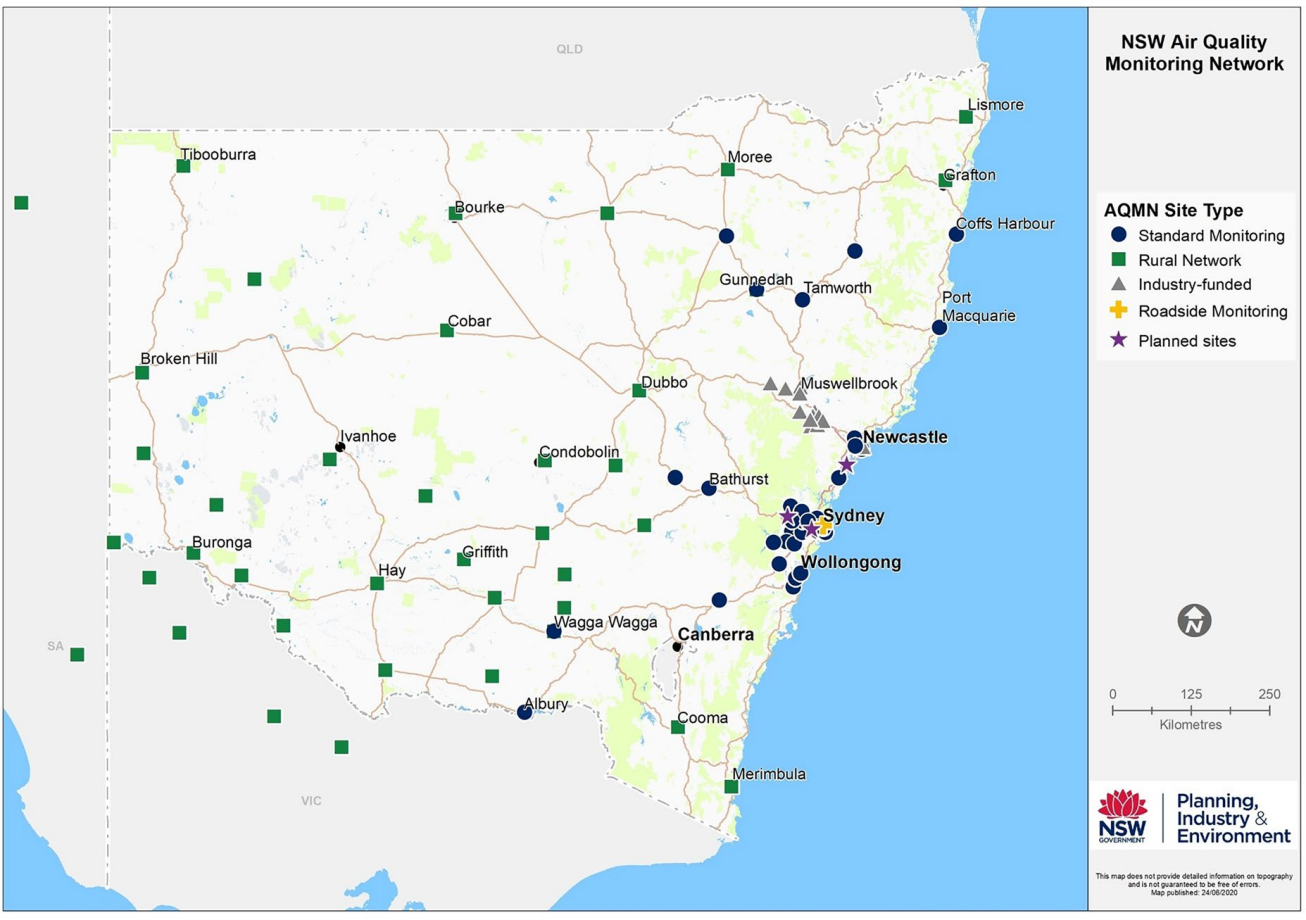
Locations of monitoring stations in the New South Wales Air Quality Monitoring Network, as of mid-2020. Standard-compliant method instruments for PM_2.5_ are applied at the standard, industry-funded and roadside monitoring stations

PM_2.5_ pollution can be generated naturally or from human activities. In New South Wales (NSW), for example, typical natural sources of PM_2.5_ include vegetation fires, dust storms, pollen and sea sprays, whereas major human sources are transport, power generation, domestic activities, and commercial and industrial processes (NSWEPA, 2019). In addition, secondary particles can be formed in the atmosphere through complex reactions of chemicals (pollutants) such as sulfur dioxide and oxides of nitrogen (Cope et al., 2014). The Australian National Environment Protection (Ambient Air Quality) Measure (NEPM hereafter) sets out the national standards for PM_2.5_ (and PM_10_) pollution, as well as its monitoring and reporting protocols, for Australian jurisdictions (NEPC, 2011; NEPM, 2021). In this context, the present study is concerned with the validity of negative hourly PM_2.5_ readings from 30 standard monitoring stations in the NSW Air Quality Monitoring Network (AQMN; Fig. 1; Riley et al., 2020), which are reported against the NEPM in NSW annual compliance reporting (e.g. DPIE, 2021).

Beta Attenuation Monitors (BAM), one of the United States (US) Environmental Protection Agency (EPA) Federal Equivalent Methods for PM_2.5_ measurements (USEPA, 2016a), are commonly used for ambient PM_2.5_ compliance monitoring in Australian jurisdictions (Standards Australia, 2022a), as is in many other countries. BAM instruments adopt an optical measurement technique (Chung et al., 2001). They draw sample air through a size selective inlet and onto a filter tape where PM_2.5_ is collected. Beta radiation passing through the filter tape is measured—it reduces over time when more particles are deposited onto the tape. The change in beta radiation is proportional to the mass of particulates added to the tape, thus determining the PM_2.5_ measurements (Scientific, 2018).

In real-world situations, ambient air always contains certain amounts of particles (and other pollutants), and negative PM_2.5_ concentrations should never occur. In the monitoring practice, however, instruments such as BAM can and do occasionally record negative PM_2.5_ measurements. For example, when air is relatively clean (e.g. on a day after some good rain) and PM_2.5_ levels are very low, falling in the range of the instrument detection limit, the PM_2.5_ readings may be either slightly positive or negative, but with long-term (over many hours) averages reflective of actual low PM concentrations. The instruments are designed with mechanisms such as dryers or heating elements to account for the impact from volatile partitioning, (to some degree) suppressing the chance for obtaining negative measurements (Scientific, 2018). Nevertheless, when taking dynamic air samples across short time periods, the instruments can still give rise to positive or negative measurement spikes of significant amplitude, usually on hourly or sub-hourly time scales.

A common approach for dealing with negative particle data, especially those unrelated to instrument malfunctions or procedural errors, is to set a threshold of negative value, above which the negative measurements are retained as valid in the data system, but below which the negative values are invalidated (or flagged for further investigation) and excluded from subsequent calculations and data reporting. It is apparent that this validation approach has a risk of causing *significant* (positive) bias in compliance reporting if the threshold is set to an inappropriate level, e.g. at zero concentration.

The NSW AQMN employs the Thermo Scientific BAM (5014i Beta, and previously the 5030i Sharp) instruments for PM_2.5_ monitoring at the NEPM stations (i.e. standard stations in Fig. 1), with a small number of TEOM-FDMS (TEOM 1405-DF) monitors applied at some other stations (Scientific, 2018; Standards Australia, 2022b; Thermo Scientific, 2009, 2014). The NSW AQMN holds accreditation by the National Association of Testing Authorities (NATA) for the methods and operating procedures for PM_2.5_ data collection with these instruments. In this context, the cause for negative data or how to reduce the number of negative values is not discussed in the present study, with such an investigation to be undertaken elsewhere.

We propose that the threshold for controlling negative PM_2.5_ readings, especially those unrelated to instrument malfunctions or procedural errors, should be determined with consideration of factors such as measurement uncertainties, instrument noise and implications for air quality reporting. This paper demonstrates how the *threshold value* is determined for controlling negative hourly PM_2.5_ measurements from the BAM instruments in the NSW AQMN. It involves a literature review of thresholds used by different agencies (data systems), followed by an assessment of the instrument measurement uncertainties, zero (clean) air test data distribution and impacts on three key NEPM compliance reporting statistics (data availability rate, annual average level and number of exceedance days) when applying different thresholds. It is notable that we have also conducted a similar study on the control of negative PM_10_ measurements from TEOM instruments, with the results to be presented elsewhere.

The initial project output has served to be part of local evidence for formulating the recently published Australian Standard, AS 3580.19.2020 – Method 19: Ambient air quality data validation and reporting (Standards Australia, 2020). The analysis results are expected to be useful for the air quality data management and reporting practice in other Australian jurisdictions or wider contexts. In the following sections, an overview of the analytic framework and data processing method is given in the “Analytic framework and data processing” section, then the results and discussions are presented in the “Results and discussions” section, and finally the summary of findings and conclusions are provided in the “Summary and conclusion” section.

## Analytic framework and data processing

### Analytic framework

**Fig. 2:**
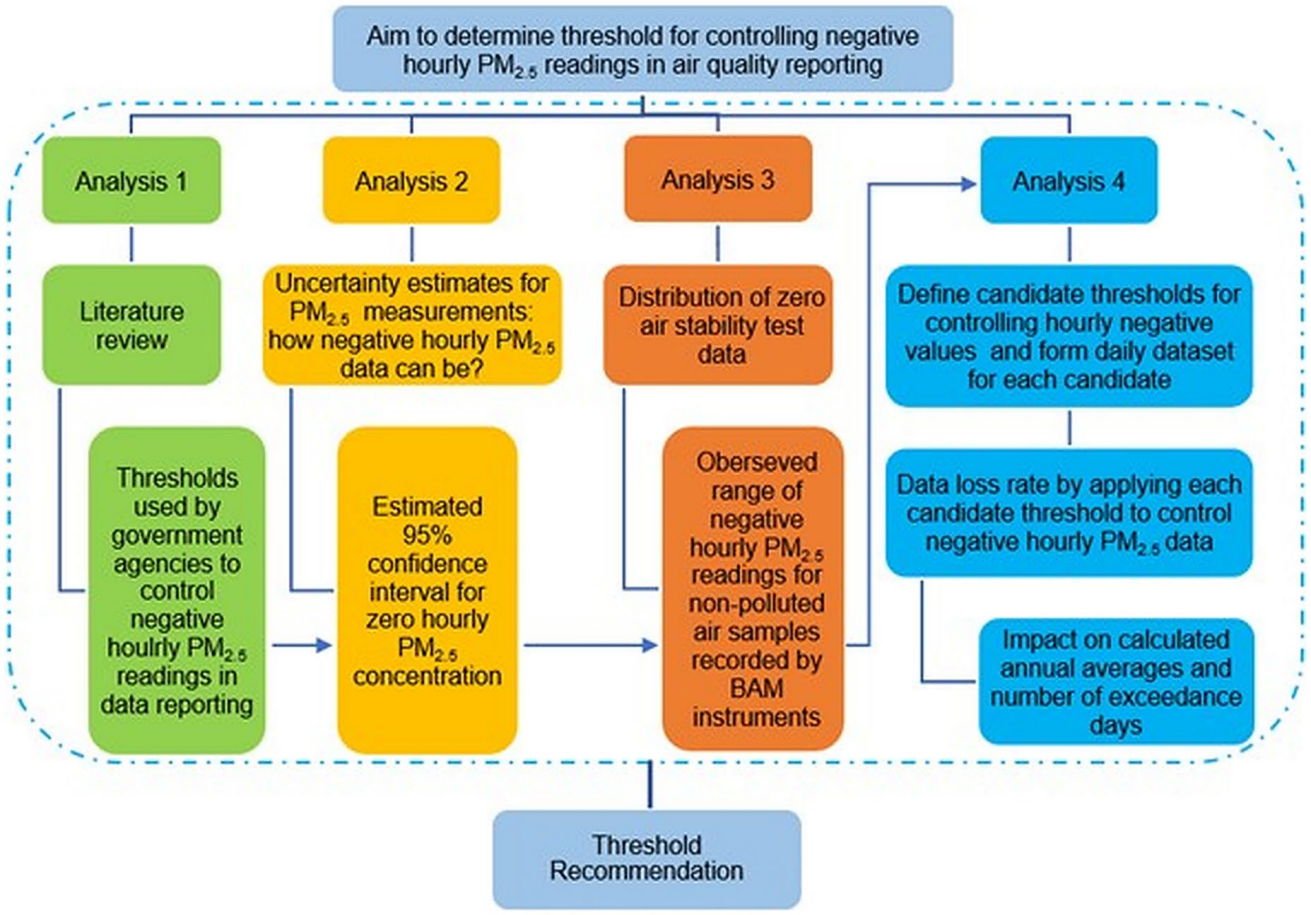
Schematic of the analytic framework for the study

The study was undertaken within a framework of four inter-related analyses (Fig. 2). Analysis 1 was to provide a brief overview of thresholds adopted in other data systems for controlling negative PM_2.5_ readings in data reporting. Analysis 2 was to discuss the theoretical estimates of measurement uncertainties for PM_2.5_ data in the NSW AQMN. The expanded measurement uncertainties were calculated as a function of measured (zero or positive) hourly PM_2.5_ concentrations of ambient air samples, following the calculation method outlined in JCGM (2008), “Evaluation of measurement data-Guide to the expression of uncertainty in measurement” (the GUM). The calculation included the component uncertainties associated with major operating elements in data collection, such as instrument calibration and maintenance, instrument operation, flow rate, and operating temperature and pressure. Analysis 3 was to examine the distribution of the zero (clean) air test data for BAM instruments in the NSW AQMN (also the “Zero air stability test data” section), to identify the observed range of positive and negative measurements for non-polluted air samples, due to impacts of factors such as detection limit, instrument noise and procedural uncertainty. Analysis 4 was to assess the impacts of the threshold choice to control negative PM_2.5_ data on three key NEPM reporting statistics, i.e. data availability rate, annual average and annual number of exceedance days (details in DPIE (2021)) by applying individual candidate thresholds to the historical PM_2.5_ data from the NSW AQMN (“PM_2.5_ data” section). The candidate thresholds were specified by drawing upon outputs from Analyses 1 to 3. At last, integrating the outputs from four analyses, the operational threshold for controlling relatively large negative hourly PM_2.5_ readings has been recommended for the NSW AQMN data system that supports air quality compliance reporting.

### Zero air stability test data

In the NSW AQMN, zero air stability test is undertaken annually for each BAM instrument, where the instrument is run with a zero-particulate filter (a filter that removes particles from the sampled air) on the sampling inlet, to examine instrument stability and operating issues under particle-free air.

The zero air stability test data were obtained as 1-min readings from BAM instruments at 30 stations for a period of 8–26 h annually during 2013–2017 (further details in Appendix Table 2 and Fig. 1). The 1-min data relating to instrument faults or procedural errors were excluded from further calculations. Hourly average was derived if the availability rate of 1-min data was ≥75% for that hour. The hourly data were pooled for all stations to form datasets by year and all-year combined. These data were used for Analysis 3, to examine the observed range of positive and negative measurements for non-polluted air samples, due to impacts of factors such as detection limit, instrument noise and procedural uncertainty.

### PM_2.5_ data

Hourly PM_2.5_ concentration data, including positive (valid) or negative values, were obtained for 2013–2017 from the same 30 stations in the NSW AQMN. Most stations had data for near 5 years, with a small subset commencing PM_2.5_ monitoring post 2013 (details in Appendix Table 2). The initial hourly PM_2.5_ dataset were cleaned by excluding negative data associated with identifiable instrument faults or procedural errors. Five analysis datasets were then generated by applying separately five candidate thresholds, ranging from −2.5 to −20.0 μg/m^3^, to control large negative hourly records. In each dataset, negative data were invalidated if values fell below the relevant threshold.

The Australian NEPM reporting protocols require each jurisdiction to report data availability rate, annual average and number of exceedance days for each station in the annual compliance report (e.g. DPIE, 2021). In this study, we calculated the data loss rate (%) as proxy to the data availability rate for each station and year when applying one of the candidate thresholds. The higher the data loss rate, the lower the data availability rate. Daily and annual averages were calculated for each site and year from validated datasets, if the minimum data availability rate of 75% was met for that site and year (DPIE, 2021). The number of exceedance days was calculated as the total number of days when daily average PM_2.5_ levels exceeded the national standard (at the time) of 25 μg/m^3^ for 24h PM_2.5_ in each year. The annual averages were calculated from daily averages and compared to the national standard (at the time) of 8 μg/m^3^ for annual mean PM_2.5_.

## Results and discussions

### The review—how negative PM_2.5_ readings are treated

There appears to be limited documentation on methods for handling negative PM_2.5_ measurements in the air quality literature. This review, by no means exhaustive, covered some BAM instrument manuals, Australian standards for continuous PM_2.5_ monitoring, and published documents or reports for government-operated data systems in the USA, the UK, New Zealand and locally Australia. This section describes the main findings from the review.

Air quality information end users, including general citizens, community group members and air quality managers, often hold a perception that ambient air pollution can be high or low but never go negative (Dennis, 2013; Standards Australia, 2022a, 2022b). This perception acknowledges the physical existence of pollutants in the ambient air, often with little or no consideration of impacts of procedural and technological limitations, and/or uncertainties in measurement techniques. Therefore, it is generally agreeable that negative data should be excluded in public reporting (USEPA, 2014, 2016b).

In the real-world monitoring practice, some negative readings are caused by instrument faults or procedural errors, thus can be simply invalided (flagged) and excluded from air quality reporting towards the public domain. There are occasions, however, when negative PM.5 readings occur due to other (less apparent) factors, such as instrument uncertainties, noise associated with sampling air flow, detection limit and corrections made within the instruments (associated with inlet and ambient temperature and humidity) (Scientific, 2018; Standards Australia, 2020; Thermo Scientific, 2009, 2014). For example, Met One (2021) noted that if the actual ambient particle level is below the instrument detection limit, instruments may report PM_2.5_ measurements as being slightly negative or positive. It is also known that during the measurement process, volatiles may partition to or from particles, and subsequently either increase or decrease the actual particle mass within the system, (sometimes) causing intensified short-term *positive or negative* fluctuations (spikes) in PM_2.5_ readings. In principle, the negative concentrations should be treated as valid, because invalidating them or simply correcting them to zero would lead to artificial positive bias when calculating data averages (Dennis, 2013; Met One, 2021). Of note is that, practically, positive artefact readings are often of less concern in data reporting, since PM_2.5_ concentrations are generally above zero level in most urban cities in the world, particularly at those locations selected by government agencies for monitoring population exposure to air pollution.

Some (limited) guidance is given in Australian standards, for example, AS/NZS 3580.9.13, the standard for “Determination of suspended particulate matter PM_2.5_ continuous direct mass method using a tapered element oscillating microbalance monitor” (Standards Australia, 2022b). For TEOM, it is reasonable to expect some small hourly negative values when the true mass concentration is very low, for example, during rain events. As general guidance, small negative values (if within instrumental uncertainty) should be considered *clean* air conditions, i.e. being treated as valid for data reporting. When used to produce daily averages, all valid hourly values (both positive and negative) should be averaged using equal weighting, and the daily averages calculated from hourly averages should be non-negative in general, at most slightly negative but within manufacturer’s specified tolerance.

In air quality data management, a common practice is to set a threshold of negative value, under which negative data are invalidated and excluded in further calculations but with minimal risk of causing significant bias for data reporting. PM monitor manufactures usually specify allowable negative readings in their instrument user manuals with reference to the instrument detection limit (Dennis, 2013). It is not uncommon for the field zero (clean) air test to result in a value which is several micrograms different from the factory-set value (positive or negative), because the factory zero air test may be run without an inlet heater. Probably associated with this consideration, Met One (2021) noted that you should not see (in field monitoring) multi-hour periods of continuing negative concentrations, or concentration data clipped at the lower range limit of −15μg/m^3^. In contrast, some instruments, particularly those employing optical counters, pre-emptively and incorrectly truncate negative results to zero to avoid uninformed user complaints.

The US EPA has had a long-standing convention of retaining short-term (hourly) averaged negative data in its air quality databases, such as Air Quality System (AQS) and AirNow (USEPA, 2014). The AQS database treats negative data from PM_2.5_ continuous monitors as valid if the values are not below the threshold of −10.0 μg/m^3^. The AirNow PM_2.5_ database flags the readings invalid when the values are below the threshold of −4.99 μg/m^3^.

The UK Automatic Urban and Rural Monitoring Network applies ongoing data validation as part of its normal process to “clean-up” the initial provisional data (DEFRA, 2016). Corrections to data made during the validation process are automatically uploaded online for end users to access. In relation to PM_2.5_ data, it was noted that isolated or occasional negative values are permitted to remain in the dataset so long as they are not below the lower bound of the detection limit or measurement uncertainty of the method.

Ministry for the Environment (MfE), New Zealand, recommended that unless there is good evidence to remove a positive or negative value, it should be treated as valid, and comment made in the metadata (MfE, 2009). There are two treatments worth highlighting. Firstly, where negative values are within the expected error of the instrument, they should be retained within the dataset to avoid creating a positive bias in data reporting. Secondly, where large negative spikes are observed in the data record, there is a need to check whether a large positive spike is also present—if both a large positive and a large negative spike are present, both spikes should be treated invalid and removed. Such large spikes are noted as artefacts in techniques which use oscillating components, such as TEOMs. However, there is no information on the choice of threshold values used to control hourly negative PM_2.5_ data in that process. There is ongoing discussion in Aotearoa, New Zealand, on how to manage negative values from BAMs, with no definitive or agreed method used by operators (Coulson et al., 2021).

In Australia, the threshold values adopted for validating negative PM_2.5_ data vary across jurisdictions. For example, (prior to this study) the NSW AQMN data system applied a threshold of −2.5 μg/m^3^, under which negative hourly PM_2.5_ readings were automatically (provisionally) invalidated (flagged) in an initial quality control process designed for near real-time online data reporting. In contrast, the Queensland Government (2022) air quality reporting has adopted methods varying among instrument types, with the lowest validation threshold being -5.0 μg/m^3^ set for TEOM-FDMS instruments.

In summary, it is a consensus that the negative PM_2.5_ readings, if unrelated to instrument faults or procedural errors, should be treated as valid in data calculations, however need to be controlled for the purpose of public reporting. The threshold value used for controlling negative measurements varies across data systems, but there is little or no published research on how such validation thresholds are determined by the agencies. Drawing upon these findings, next we present analytic results from our study to demonstrate how a *threshold value* has been chosen for validating negative hourly PM_2.5_ measurements from the BAM instruments in the NSW AQMN.

### Measurement uncertainty estimates

**Fig. 3:**
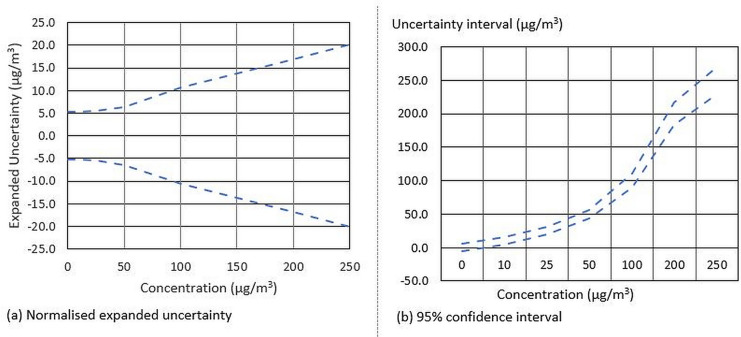
Expanded uncertainty (**a**) and 95% confidence interval (**b**) for hourly PM_2.5_ measurements

Measurement uncertainties were calculated for PM_2.5_ data collection with BAM instruments in the NSW AQMN, following the GUM procedure outlined in JCGM (2008). The uncertainty estimates included impacts from the major quantifiable uncertainty component items, such as sampling head efficiency, instrument precision and accuracy, temperature and pressure. The estimates are multiplied by a factor of 2 to arrive at the expanded uncertainties (i.e. 95% confidence limit), approximating the *t*-value associated with a normal population at *p*=0.05, as shown in Fig. 3.

It is notable that the expanded uncertainty estimates increase with measured PM_2.5_ concentrations (Fig. 3a). The expanded uncertainty range is around ±5 μg/m^3^ for a reading of near zero concentration from relatively clean air samples, with the estimates increasing to around ±10 μg/m^3^ for a reading of 100 μg/m^3^ from (more) polluted air samples. When the true PM_2.5_ level is slightly positive, it is possible that the instrument may report either small negative or positive readings, as is illustrated by the 95% confidence intervals at lower end of PM_2.5_ concentrations (Fig. 3b). For instance, if the true PM_2.5_ concentration is 2.5 μg/m^3^, the instrument may report a measurement in the range of (−2.5, 7.5) μg/m^3^ with 95% confidence.

### Properties of the zero (clean) air stability test data

**Fig. 4:**
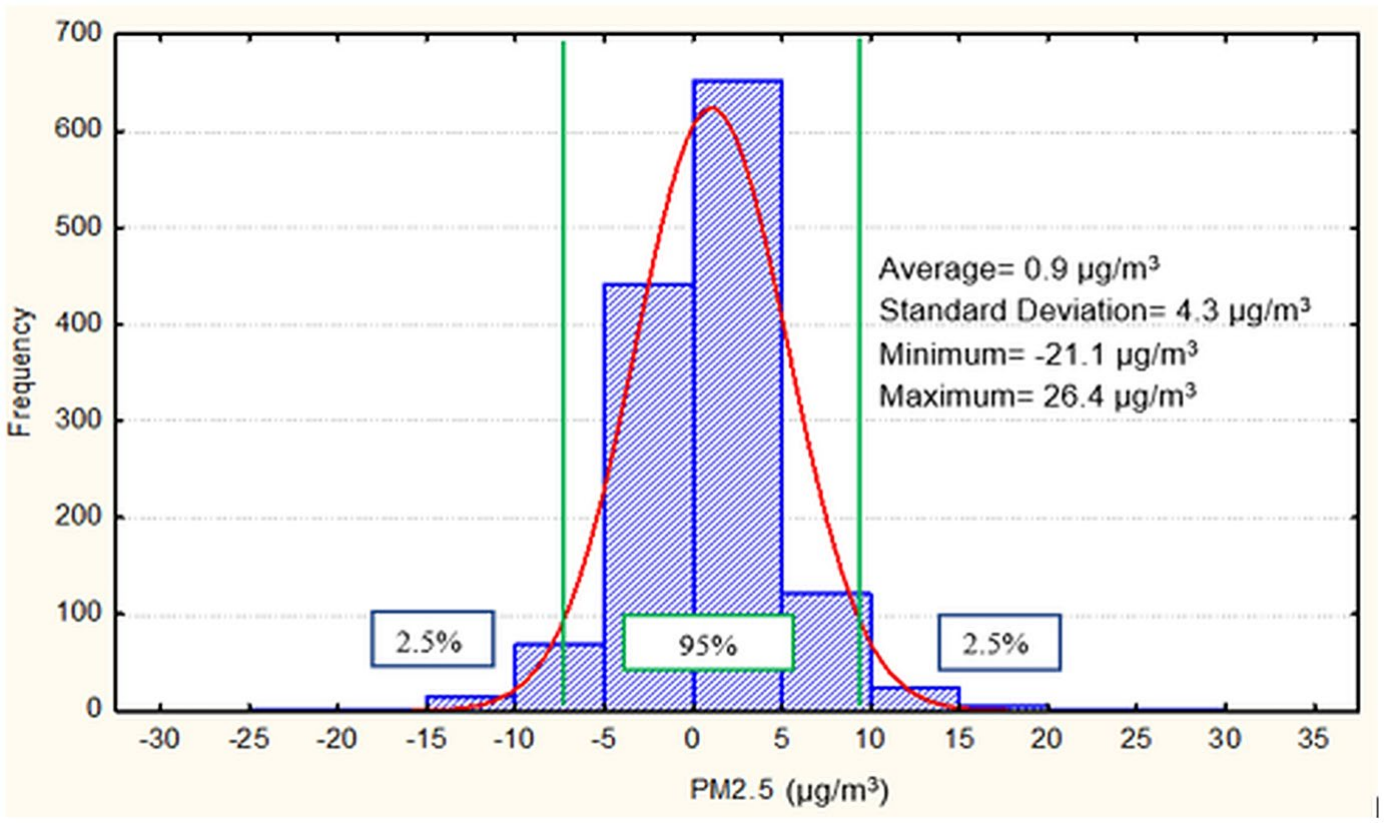
Histogram of hourly zero (clean) air stability test data, pooled for all sites and all years

An examination of the zero air stability test data helps to investigate PM_2.5_ measurement fluctuations and how negative they can be for (near) clean air samples. Table 1 shows the summary statistics for the hourly zero air stability test data by year and for all years combined, with data pooled for all stations in 2013–2017. The annual mean hourly readings across years tended to be slightly positive, around 0.9 μg/m^3^ on average. The standard deviation is 4.3 μg/m^3^ for the combined data, but varying from 2.2 to 5.1 μg/m^3^ for datasets across years. Of note is that maximum and minimum hourly averages could occasionally be significantly deviated from zero concentration level, reaching as high as 26.4 μg/m^3^ (maxima in 2014) and as low as −21.1 μg/m^3^ (minima in 2015). Figure 4 illustrates the overall distribution of hourly zero air test data, pooled for all sites and all years. The majority of hourly data fall within the range of −5.0 to 5.0 μg/m^3^, with 95% of hourly data falling into the range of −7.6, 9.4 μg/m^3^.

**Table 1:** Summary statistics for hourly zero (clean) air stability test data pooled for all sites by year and for all years combined in 2013–2017

Year	Number of hours	Mean (μg/m^3^)	Standard deviation (μg/m^3^)	Maximum hourly average (μg/m^3^)	Minimum hourly average (μg/m^3^)
2013	25	−0.7	2.2	2.8	−7.1
2014	216	1.2	4.8	26.4	−13.4
2015	305	0.5	5.1	18.4	−21.1
2016	418	1.3	3.9	20.0	−10.9
2017	368	0.7	3.7	17.9	−11.1
All years combined	1332	0.9	4.3	26.4	−21.1

Of note is that the summary statistics show some degree of variability across regions, with slightly higher mean and larger range in zero air readings for the Sydney stations if compared to stations in other regions. This could be due to impacts of factors such as variability in meteorological conditions across monitoring sites. USEPA (2016b) examined the relationship between ambient dewpoint and zero test results for Met One BAM 1020 applied in the USA, showing that the magnitude of the zero response is somewhat variable but has an overall reverse relationship with dewpoint. This aspect will be further examined in our future work for identifying potential environmental and procedural causes of negative PM_2.5_ data and thereby methods to reduce the number of negative readings in the NSW AQMN.

The deviation of measurements from zero air is essentially related to experimental and measurement uncertainties or limitations. These include instrument noise, detection limit, deposition or volatilisation of semi-volatile components (between aerosol and gas phases depending on heating cycle), and bias due to small particles passing through the filters used to generate zero air and hence leading to slightly positive results. The deviation is consistent with the GUM uncertainty estimates described in the “Measurement uncertainty estimates” section, with the zero air test data showing larger range of fluctuations between negative and positive readings. The difference is expected since the GUM calculation method covers only some (rather than all) of the quantifiable impacts for selected uncertainty elements, with influences from factors such as volatile partitioning not being considered.

The amplitude of negative readings from BAM instruments in the NSW AQMN appears consistent with overseas results. For example, the manufacturer of the Met One BAM monitors suggests allowable hourly averaged negatives as low as −15.0 μg/m^3^ (Met One, 2021). In conclusion, the BAM PM_2.5_ readings for zero air samples fluctuated around zero concentration, with good chance for going negative significantly below the threshold of −2.5 μg/m^3^ that was previously set in the NSW AQMN data system for controlling negative hourly data.

### Impact on key compliance reporting statistics

The “Measurement uncertainty estimates” and “Properties of the zero (clean) air stability test data” sections have demonstrated that BAM instruments reported negative hourly PM_2.5_ measurements as low as −21.1 μg/m^3^ for zero air samples. Hence, a set of thresholds, i.e. −2.5 μg/m^3^, −5.0 μg/m^3^, −10.0 μg/m^3^, −15.0 μg/m^3^ and −20.0 μg/m^3^, was chosen as candidates for validating hourly PM_2.5_ data from the NSW AQMN for the purpose of air quality reporting. The existing threshold of −2.5 μg/m^3^ was included in the analysis as benchmark for comparison of results. The following subsections compare the impacts of different threshold options on three key NEPM compliance reporting statistics, i.e. availability rate, annual average concentration and number of exceedance days (DPIE, 2021; NEPM, 2021).

#### Impact on data availability

When a candidate threshold is applied to the dataset for a monitoring station, the hourly data availability rate changes, as do the statistics derived from hourly averages. Under the Australian NEPM monitoring and reporting protocols (DPIE, 2021; NEPM, 2021), for a daily average PM_2.5_ measurement to be valid at a monitoring station, at least 75% of the underlying hourly data must be valid (note: the data availability requirement is equivalent to similar standards applied in North America, Europe and China). Application of a threshold can potentially invalidate too many hours of records which are within the range of experimental noise or detection limit and hence in fact valid measurements.

**Fig. 5:**
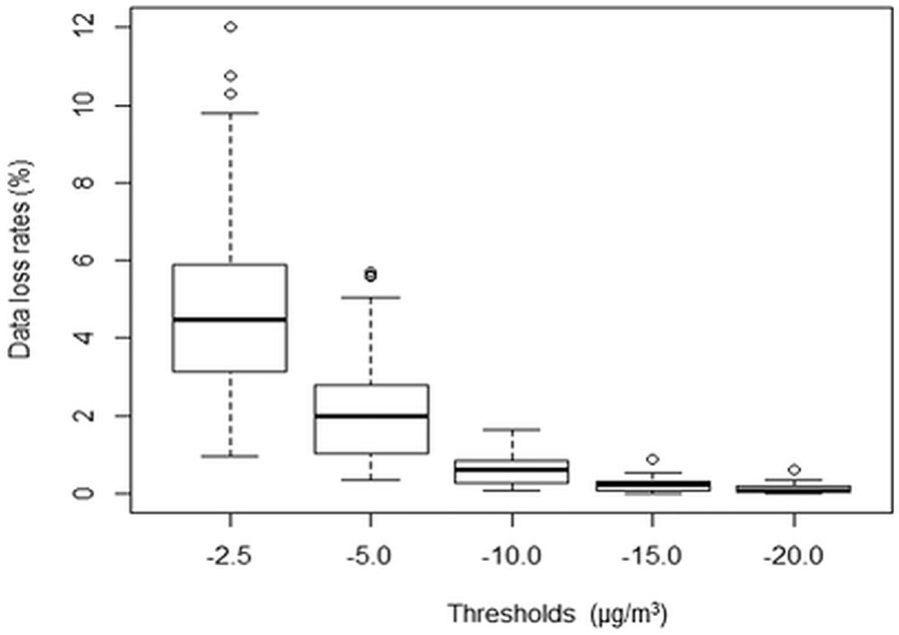
Box plot of annual data loss rates (%) for individual stations and years by threshold option. Bold line inside box: median; box bottom and top sides: 25th and 75th percentiles; circle: outliers; lines outside the box: 9th and 91st percentiles

Figure 5 shows the box plot of the data loss rates (“PM_2.5_ data” section) by different threshold options for all sites from 2013 to 2017. The data loss rate is expressed as percentage of negative hourly readings invalidated due to application of the chosen threshold for each site and year. The higher the data loss rate, the higher the reduction in data availability rate, due to the choice of validation threshold. As expected, the data loss rates show a decreasing trend when the threshold applied becomes more negative, where the inference can be made in a way analogous to application of a scree-test. The data loss rates are the largest on thresholds −2.5 μg/m^3^ (inter-quartile range: around 2.6–6.0%), deceasing quickly through threshold −5.0 μg/m^3^ (inter-quartile range: around 1.2–2.4%) till threshold −10.0 μg/m^3^ (inter-quartile range: located below 1.0%). The change in data loss rate flattens from threshold −10.0 μg/m^3^ towards more negative values. In other words, the choice of −2.5 μg/m^3^ or −5.0 μg/m^3^ as validation threshold has more significant impacts on data availability rates, if compared to the choice of −10.0 μg/m^3^ or lower values. With an intention of keeping data loss rates as low as possible (e.g. under 1%), the threshold of −10.0 μg/m^3^ appears to be a good compromise for controlling the release of negative hourly readings in public reporting.

#### Impact on calculated annual averages

**Fig. 6:**
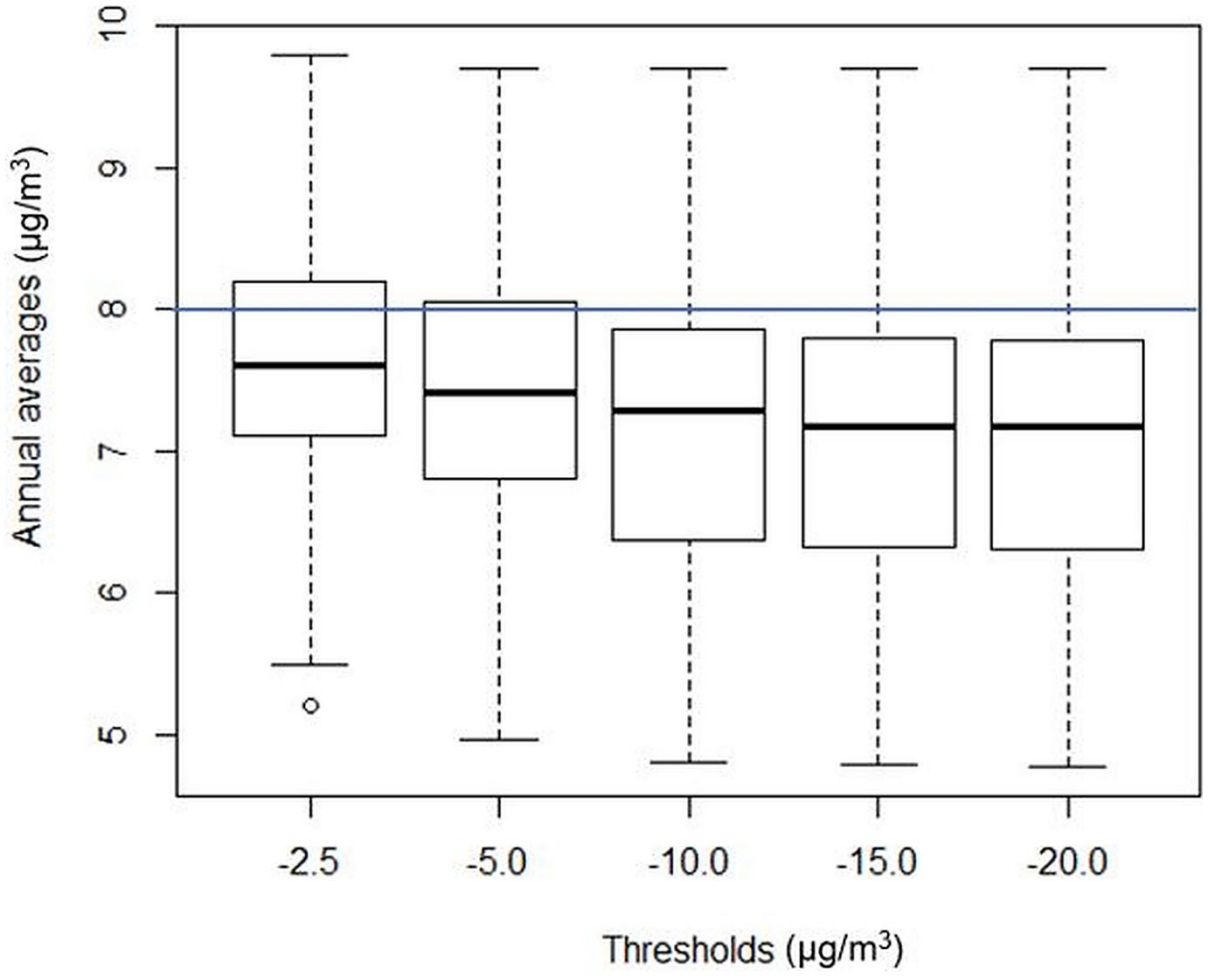
The box plot of annual averages for individual stations and years by threshold option. Bold line inside box: median; box bottom and top sides: 25th and 75th percentiles; circle: outliers; lines outside the box: 9th and 91st percentiles

From the previous section, the data availability rate changes with the choice of validation threshold, and therefore, the annual PM_2.5_ averages are expected also to decrease if a more negative hourly threshold is applied. Figure 6 shows that the box plot of annual averages for individual sites in 2013–2017 by threshold options. In general, the smaller the amplitude of validation thresholds, the larger the site annual averages and subsequently the higher chance for exceeding the NEPM national standard of 8.0 μg/m^3^.

In particular, the candidate thresholds of −2.5 μg/m^3^ and −5.0 μg/m^3^ are associated with slightly larger median values and higher box locations, if compared to the threshold of −10.0 μg/m^3^ or lower (which show generally similar data distributions). In particular, the inter-quartile ranges for the former tend to overlap over the national standard, implying a potential for positive bias in reporting annual PM_2.5_ data due to control for negative readings. In contrast, the threshold of −10.0 μg/m^3^ appears to a middle-way choice with reduced chance for biased reporting of annual exceedance.

#### Impact on the number of exceedance days

The NEPM standard for daily average PM_2.5_ is currently 25.0 μg/m^3^. The number of exceedances was calculated from daily averages. The greater the negative thresholds, the high number of negative hourly values included in the dataset and subsequently the lower the daily average concentrations. The number of exceedance days was examined by threshold option, at every site and year in 2013–2017.

It is found that the exceedance days are identical across different validation thresholds, for each specific site and year (not shown). In other words, the choice of different thresholds does not affect the reporting of annual exceedance days for monitoring stations in this network for the examined dataset. This lack of change in the number of exceedance days is not surprising. Days on which the daily average PM_2.5_ is approaching or exceeding 25.0 μg/m^3^ have very little chance with hours when there are negative values, because the experimental noise is not sufficiently large to drop below the existing threshold due to higher measured (actual) pollution levels. Hence, the main priority in choosing the validation threshold is to ensure that bias for data availability rate and annual average reporting is kept minimal, since the impact on the number of annual exceedance days is generally negligible in the NSW AQMN.

## Summary and conclusion

This paper reports on an investigation on the choice of validation threshold for controlling negative hourly PM_2.5_ readings in the NSW air quality data system, for the purpose of public (compliance) reporting. It involves a brief literature review on the validation methods and thresholds used in different government data systems, followed by an assessment of the instrument measurement uncertainties, zero (clean) air test data distribution and impact of applying alternative thresholds on three key compliance reporting statistics (data availability rate, annual average concentration and annual number of exceedance days) for NSW AQMN. The main findings are summarised below:

- It is generally agreeable that valid negative numbers should be included in air quality databases but excluded in public reporting. The choice of threshold values for validating negative hourly PM_2.5_ data varies across data systems, with little research reported in the literature on how these thresholds are determined by the agencies.
- The theoretical estimates of measurement uncertainty increase with the level of PM_2.5_ concentrations, with the uncertainty for (near) zero concentration reading being in the range of (−5, 5) μg/m^3^ at the 95% confidence.
- The NSW AQMN zero air stability test data have revealed that the hourly PM_2.5_ readings for (near) clean air samples can go below zero, as negative as −21.1 μg/m^3^ in 2013–2017, with 95% hourly zero air PM_2.5_ measurements falling in the range of (−7.6, 9.4) μg/m^3^.
- The choice of thresholds has distinguishable impacts on data availability rates and annual average concentrations. Of the threshold options examined, the impact is more significant for thresholds of smaller amplitude, such as −2.5 μg/m^3^ and −5.0 μg/m^3^. In contrast, the choice of thresholds makes no difference in calculating the annual number of exceedance days for individual stations for the years examined.
- The threshold of (near) −10.0 μg/m^3^ appears to be the optimal value for controlling negative PM_2.5_ data release in public reporting. This threshold is consistent with the measurement uncertainty estimates and zero air test data statistics calculated for NSW AQMN, and also is expected not to have significant impacts on key compliance reporting statistics such as data availability rates and annual averages.

Prior this study, the validation threshold was set to −2.5 μg/m^3^ in the NSW AQMN, below which the negative readings were invalided or flagged for further investigation. This study has indicated that the use of such a threshold could potentially result in distinguishable bias in reporting statistics such as data availability rates and annual averages. Hence, we have made the following recommendations:

A) The validation threshold is set to −10.0 μg/m^3^ to control the negative hourly PM_2.5_ data in the NSW AQMN data system, under which the hourly negative readings are invalidated/flagged for further investigation.
B) Those negative readings due to instrument faults or procedural errors can be simply invalided (flagged) and excluded from subsequent calculations for data reporting.
C) Other negative values should be reviewed during the data validation process to assess whether they are real or spurious. Unless there is good evidence to remove a value, it should be left as a valid reading.
D) When large negative spikes are observed, check also to see whether a large positive spike is present. If both a large positive and a large negative spike are present and sequential, both spikes should be treated as invalid data and the cause be investigated.

This work has facilitated a variation in control of negative data in the NSW AQMN data system. It also served to be part of local evidence for formulating of the published Australian Standard for data validation and reporting (Standards Australia, 2020). The results are expected to be useful for air quality data management and reporting in other jurisdictions in Australia or wider contexts. An investigation of the cause for negative data or how to reduce the number of negative values is to be discussed elsewhere.

## Data Availability

The datasets generated and/or analysed during the current study are available from the corresponding author on reasonable request.

## Appendix

**Table 2:** BAM instrument model and data periods by monitoring site

Region and site	Year with 1-min zero air stability test data	Time period with hourly ambient PM_2.5_ data	BAM model applied
Sydney region (14 sites)			
Camden	2014–2017	1/01/2013 to 31/12/2017	BAM 5014i
Chullora	2013–2016	1/01/2013 to 31/12/2017	BAM 5014i
Earlwood	2014–2016	1/01/2013 to 31/12/2017	BAM 5014i
Liverpool	2014–2017	1/01/2013 to 31/12/2017	BAM 5014i
Richmond	2014–2017	1/01/2013 to 31/12/2017	BAM 5014i
Rozelle	2015, 2016	16/02/2015 to 31/12/2017	BAM 5014i
Prospect	2015, 2016	16/10/2015 to 31/12/2017	BAM 5014i
Campbelltown West	2016, 2017	16/09/3015 to 31/12/2017	BAM 5014i
St Marys	2016	14/03/2016 to 31/12/2017	BAM 5014i
Bringelly	2016	27/06/2016 to 31/12/2017	BAM 5014i
Oakdale	2016, 2017	1/01/2013 to 31/12/2017	BAM 5014i
Bargo	2017	2/12/2016 to 31/12/2017	BAM 5014i
Macquarie Park	2017	1/08/2017 to 31/12/2017	BAM 5014i
Randwick	2016, 2017	23/03/2017 to 31/12/2017	BAM 5014i
Illawarra region (3 sites)			
Albion Park South	2016	26/02/2015 to 31/12/2017	BAM 5014i
Kembla Grange	2015–2017	25/02/2015 to 31/12/2017	BAM 5014i
Wollongong	2015, 2016	1/01/2013 to 31/12/2017	BAM 5014i
Lower Hunter region (6 sites)			
Beresfield	2013–2017	1/01/2013 to 31/12/2017	BAM 5014i
Wallsend	2014–2017	1/01/2013 to 31/12/2017	BAM 5014i
Newcastle	2014–2016	18/08/2014 to 31/12/2017	BAM 5014i
Mayfield	2014–2017	15/07/2014 to 31/12/2017	BAM 5014i
Carrington	2015–2017	31/07/2014 to 31/12/2017	BAM 5014i
Stockton	2014–2017	15/10/2014 to 31/12/2017	BAM 5014i
Central coast region (1 site)			
Wyong	2014–2017	1/01/2013 to 31/12/2017	BAM 5014i
Upper Hunter region (3 sites)			
Camberwell	2014–2017	1/01/2013 to 31/12/2017	Sharp 5030
Singleton	2015–2017	1/01/2013 to 31/12/2017	Sharp 5030
Muswellbrook	2014–2017	1/01/2013 to 31/12/2017	Sharp 5030
Rural NSW (4 sites)			
Albury	2017	14/09/2016 to 31/12/2017	BAM 5014i
Bathurst	2016	23/04/2016 to 31/12/2017	BAM 5014i
Tamworth	2015, 2016	13/08/2015 to 31/12/2017	BAM 5014i
Wagga Wagga North	2014–2017	1/01/2013 to 31/12/2017	BAM 5014i
